# Therapeutic Notes

**Published:** 1896-10-17

**Authors:** 


### Therapeutic Notes.

### XEROFORM.

Xeroform is a yellowish, neutral, and insoluble powder. It appears to have no toxic qualities, and is not readily decomposed by heat or light into its components, oxide of bismuth and tribromphenol. On the other hand, it becomes decomposed by the action of strong acids and alkalies, and in contact with the living tissues and juices of the animal body; and, owing to this latter property, like the allied bodies salol, airol, aristol, &c., it probably owes its antiseptic properties. Xeroform first came into prominent notice during the epidemic of cholera at Hamburg, when it was extensively used by Hueppe ("Berl. Klin. Woch.," 1893, s. 162), and regarded by him almost in the light of a specific against the cholera bacillus. Laboratory experiments ("Draër Centralblat. fur Bacteriolog u Parasitkund," 1893, No. 7) have not, however, confirmed this belief. In fact, Xeroform, in its natural condition, does not appear to exercise any very powerful bactericidal properties; it is only when in contact with living tissue, and decomposed into the oxide of bismuth and tribromphenol that this property becomes manifest. The latter body is undoubtedly a powerful antiseptic, and the oxide of bismuth is by some observers believed to enter into insoluble combination with albumen, albuminoids, alkaloid, and ptomains, and hence its value in cholera, and other intestinal conditions where dangerous toxines or albumoses are formed. In the treatment of open wounds a body such as Xeroform has many obvious advantages, since by the constant liberation of a free antiseptic, where it comes in contact with living tissue, the wound itself can be preserved almost indefinitely from the injurious effects of septic bacteria.

The chief uses of Xeroform, as at present reported, appear to be internally for septic conditions of the bowel, as in cholera; for ulcers, for many forms of eczema, and other skin conditions, and for all sorts of wounds, recent and otherwise. The powder may be dusted on, or applied by means of an atomiser. Xeroform gauze may now be obtained, and may be sterilised by heat without undergoing decomposition. Xeroform may also be applied in the form of paste or ointment, but being entirely insoluble in fats of all sorts, in this way it has less opportunities for exercising its bactericidal qualities.

